# An evaluation of U.S. federal investments in newborn screening: successes, gaps, and future directions

**DOI:** 10.3389/fpubh.2026.1729659

**Published:** 2026-02-11

**Authors:** Melissa Raspa, Rebecca Wright, Sara M. Andrews, Suzanne Dolina

**Affiliations:** RTI International, Center for Genomics and Applied Public Health, Durham, NC, United States

**Keywords:** genetics education, health outcomes, newborn screening, program evaluation, public health policy

## Abstract

**Introduction:**

Newborn screening (NBS) is a successful public health program conducted by states that provides screening, confirmatory testing, and access to treatments for millions of babies each year. Federal legislation has outlined activities to support the newborn screening system. This paper summarizes an evaluation of investments made by the Health Resources and Services Administration’s (HRSA) within the U.S. Department of Health and Human Services in the newborn screening system.

**Methods:**

A total of 52 participants took part in either an interview or focus group. Participants represented a variety of NBS groups, including federal program grantees, state public health departments, healthcare providers, parents and patient advocacy representatives, newborn screening researchers, and subject matter experts. Data collection sessions were recorded and transcribed. A rapid turnaround analysis approach was used to code the qualitative data.

**Results:**

Participants provided feedback on the progress made by the newborn screening system as a result of HRSA’s investments. Although there have been a number of successes, gaps remain. Additional support is needed in the areas of education, training, and technical assistance to enhance and expand screening capacity, conduct short- and long-term follow-up, and improve health equity and outcomes.

**Conclusion:**

Newborn screening has maintained a strong tradition as a successful public health program. Continued federal investments are needed to prepare the newborn screening system for systematic changes on the horizon.

## 1 Introduction

Newborn screening (NBS) is a long-standing public health program to identify conditions in newborns that need immediate intervention to prevent medical complications, intellectual disability, or even death. NBS is often referred to as a system of services and supports, starting with the collection of dried bloodspot specimens from infants shortly after birth, screening of infants, follow-up after out-of-range screening results, confirmatory testing, outreach to and education of parents and healthcare professionals about the system and specific conditions, and long-term data collection and surveillance activities (1). NBS also includes point-of-care screening for hearing loss and congenital heart disease. With guidance from the Advisory Committee on Heritable Disorders in Newborns and Children (ACHDNC), the U.S. Department of Health and Human Services Secretary sets forth the Recommended Uniform Screening Panel (RUSP), which lists conditions to be screened. Although the RUSP provides federal level endorsement, NBS is implemented at the state level. Each year millions of babies are screened for disorders on the RUSP and approximately 13,000 are identified and referred for diagnostic testing and treatment (2).

Much has changed since NBS became routine public health practice in the 1960s. For example, a variety of federal agencies have funded research projects, pilot studies, technical assistance and quality improvement programs, and evaluation projects in NBS; the ACHDNC has established review criteria for adding new conditions to the RUSP; new laboratory technologies have been introduced and states have expanded the number of conditions for which they screen; and patient advocacy groups and biotechnology and pharmaceutical companies have worked to provide access to early identification and treatment of rare disorders (3–5). Despite the progress that has been made, more change is on the horizon as the NBS system prepares for a future in which significantly more conditions may be nominated for the RUSP and new screening technologies are available (3, 6, 7).

Recognizing the importance of this critical public health program, the Newborn Screening Saves Lives Act of 2007 (8) and the subsequent reauthorization in 2014 (9) were enacted to provide federal support of the NBS system. The legislation called for investments across a variety of activities, including programs to: (1) enhance, improve, or expand the ability of state and local public health agencies to provide screening, counseling, and healthcare services to newborns and children having heritable disorders; (2) provide education, training, and technical assistance (TA) to lab personnel and other genetics/healthcare professionals on the implementation of state-based public health NBS programs; (3) establish, maintain, and operate a system to assess and coordinate follow-up and treatment related to congenital, genetic, and metabolic conditions; (4) improve the timeliness of NBS from specimen collection through diagnosis; and (5) develop and provide education to, and engage with, consumers (i.e., parents, families, patient advocacy groups) about screening, counseling, follow-up, and treatment to increase awareness, knowledge, and understanding of NBS and genetic conditions.

Since the passage of this legislation, the Maternal and Child Health Bureau (MCHB) within the Health Resources and Services Administration (HRSA) has provided funding to support these objectives. In addition, although not specified in the legislation, HRSA also prioritizes improving health equity and health outcomes of individuals with genetic conditions, reducing morbidity and mortality caused by genetic conditions (including congenital and metabolic disorders), and improving the quality of coordinated and comprehensive genetic services to children and their families. The focus of this paper is to evaluate progress in the NBS system as a result of HRSA-supported program activities, identify gaps in the field, and provide recommendations on where to focus short- and long-term efforts to maintain and improve NBS for the benefit of children and families.

## 2 Methods

### 2.1 Evaluation approach

Our evaluation was designed using common program evaluation frameworks (10, 11). To gather evidence about the outcomes of the HRSA-funded programs, we conducted semi-structured interviews or focus groups with representatives of the NBS system. Representatives included HRSA-funded NBS program grantees, state NBS program staff, parents of children with a condition identified through NBS, patient advocates, healthcare professionals, and NBS researchers and subject matter experts. Interview and focus group questions were organized around the six HRSA-supported program activities outlined in the federal legislation as well as health equity and outcomes, and tailored to each group. For example, parent interviews focused more on NBS education, whereas subject matter experts were probed about topics such as the timeliness of NBS. We have included the state public health department guide in Supplementary material as example. All evaluation activities were determined to be Not Human Subjects Research by the (blinded for review) IRB. Nonetheless, written informed consent was obtained from all participants prior to data collection and was reviewed verbally at the start the interview or focus group.

### 2.2 Participants

Prospective participants were identified through a combination of review of the NBS literature, patient advocacy organizations’ websites for conditions on the RUSP, known subject matter experts, and input from HRSA. Although we based recruitment on a convenience sample, we wanted to recruit a diverse group of participants. For example, we recruited state NBS program staff from different regions of the country, including smaller and larger states, as well as laboratory and follow-up staff. Healthcare providers represented a variety of specialty areas, such as genetics, pediatrics or primary care, and neurology. An assortment of RUSP conditions were represented in parent and patient advocate interviewees, and parents were from different states. Because recruitment was based on familiarity with and expertise in NBS, we did not collect demographic information on participants, apart from state of residence when applicable, or use demographic information to guide sampling.

We initially invited 54 individuals to participate, with a goal of including 9 participants per group. If an invited individual did not respond after the first email, we sent up to two follow-up emails. If the individual did not respond or declined participation, we invited another individual representing the same group. In total, we invited 67 individuals and 52 consented to participate, including: (a) NBS program grantees (*n* = 9), (b) state NBS program staff (*n* = 8), (c), parents of children with a condition identified through NBS (*n* = 9), (d) patient advocates (*n* = 8), (e) healthcare professionals (*n* = 9), and (f) NBS researchers and subject matter experts (*n* = 9). In total, participants represented 3 research or professional organizations, 8 national patient advocacy organizations, and 24 NBS staff, parents, clinicians, and NBS researchers or subject matter experts from 24 states (AK, AL, AZ, CA, CO, CT, FL, GA, IA, IL, KS, MD, MI, MN, NC, NY, OH, OK, PR, RI, TN, UT, VA, and WI).

Program grantees, state public health departments, and NBS researchers and subject matter experts were asked to participate in interviews. Parents, patient advocates, and healthcare providers were asked to participate in focus groups. Each focus group was organized and conducted by type of NBS representative. However, if an individual was unavailable to participate in a focus group, we offered an interview instead. In total, 21 individuals participated in one of six focus groups lasting 90 min, and the remaining individuals participated in interviews lasting from 60 to 90 min. Participants were offered a gift card in appreciation for their time.

### 2.3 Analysis

With permission from participants, each interview or focus group was recorded and transcribed. We used rapid turnaround analysis, which is commonly used in health services and evaluation research when there is a need to triangulate findings from multiple qualitative and quantitative data sources and quickly obtain insights needed to make timely decisions regarding strategy and practice (12). Rapid turnaround analysis is ideal for studies in which a semi-structured guide is used to obtain qualitative data on pre-determined constructs of interest (13), as was the case in this evaluation.

A pair of analysts was assigned to conduct all data collection and analysis for a given participant group. Notes were taken into a structured template that aligned with the interview or moderator guide. Transcripts were reviewed to fill in gaps in notes and extract illustrative quotations. Analyst pairs then manually coded notes to tag segments that addressed the HRSA-supported program activities. We used a collaborative qualitative analysis approach (14); for each set of notes, one analyst conducted initial coding, and the second analyst reviewed the proposed coding. The pair then met to discuss any questions or disagreements and come to consensus on the final coding. Coded segments of notes were transferred to corresponding sections of a matrix for each group. Analyst pairs then created summaries across participants within their group for each program activity. The matrices helped analysts quickly and systematically investigate similarities and differences in responses across participants and data sources, (i.e., facilitating comparison within and across groups) (15, 16). Themes from the matrices were integrated into an overall summary for each program activity.

## 3 Results

Table 1 provides an overview of the themes for each of the six activities supported through HRSA program funding. Below we share participants’ views of the progress in meeting each of the activities and HRSA’s role through programmatic investments, including facilitators, barriers, and gaps to be addressed.

**Table 1:** Thematic summary of the Health Resources and Services Administration’s (HRSA)’s investments in newborn screening (NBS) program activities and gaps to be addressed.

Legislative program activities	Summary of HRSA’s investments	Gaps to be addressed
Enhance, improve, or expand NBS programs	Facilitated states’ expansion of their screening panels by supporting the implementation of new Recommended Uniform Screening Panel (RUSP) disorders Provided training from other states with experience implementing new conditions Outsourced second-tier testing to regional testing centers Provided technical assistance on new conditions through calls, webinars, and web-based resources Provided educational materials for healthcare professionals about new RUSP conditions Created a readiness tool for states to support the addition of new conditions to their panels.	Insufficient federal guidance and funding for NBS implementation Inadequate guidance and lack of consistent policies about how quickly states should add new RUSP conditions Limited funding to support states in implementing new conditions Limited capacity of the NBS system to handle new treatments and screening technology Lack of infrastructure to enable data sharing, data standards, and harmonization across state Limited collaboration among federal agencies and funded programs
Provide education, training, and technical assistance (TA) on NBS to professionals	Strong support for education and training opportunities for lab and follow-up staff, particularly around timeliness, adding new conditions, and a data repository on quality indicators Provided TA in a variety of ways, including a web site which hosted forums and a listserv, workgroups for states NBS staff on different topics, and individualized TA (both in-person and virtual)	Need for additional training and TA for state lab and follow-up staff around topics such as data analytics, adding new conditions, long-term follow-up, and communication between lab and follow-up staff Differences in resources among states which can lead to disparities Limited training and education for healthcare professionals (e.g., primary care providers, hospital staff)
Establish follow-up and treatment for affected patients and their families	Conducted webinars and trainings on short-term follow-up Provided peer-to-peer learning across a variety of topics Created a data repository that collects data on quality indicators related to timeliness of short-term follow-up	Lack of focus on long-term follow-up Inconsistency in short-term follow-up with families after a positive screen (e.g., anticipatory training related to out-of-range screens and next steps) Variation in connecting families with psychosocial supports
Improve the timeliness of NBS from specimen collection through diagnosis	Established quality indicators and support to collect data Set standards for states	Need for maintaining timeliness standards through ongoing education, quality improvement efforts, and funding Improvement of short-term follow-up quality indicators, including the amount of time from reporting screen-positive results to the receipt of medical intervention or a confirmed clinical diagnosis
Develop and disseminate educational materials	Increased the amount, quality, and availability of educational resources for families about staff Diversity of high-quality educational materials designed to reach a broad spectrum of families	Patchworked system of resources with no centralized location for all materials and little collaboration among groups Need to continuously review and update educational materials Challenges with disseminating materials to intended audiences Limited visibility and use of developed materials
Improve health equity and outcomes	Provision of education, training, and TA to enhance state capacity and support the implementation of new conditions has led to earlier diagnosis and treatment for all children Tracking of timeliness data to evaluate progress has significantly helped to improve the speed of receipt and screening of specimens and reporting results Inclusion of medically underserved and diverse families in a needs assessment to determine the design and development of various educational materials has resulted in more inclusive resources for all families	Inequities due to states screening for different conditions, when timeliness becomes an issue, when families have challenges accessing confirmatory testing, and when families transition into clinical care and have unequal access to resources (e.g., specialized treatments, transportation, social and emotional support) Challenges in assessing outcomes due to loss to follow-up Inequities due to systematic racism and implicit bias

### 3.1 Enhance, improve, or expand NBS programs

Participants agreed that progress has been made to enhance, improve, or expand the ability of state NBS programs to screen newborns and conduct follow-up. Various federally funded programs have helped create a more efficient and proficient NBS system, with most emphasis placed on supporting labs and screening techniques. In particular, HRSA funding has facilitated states’ expansion of their ability to screen by supporting the implementation of new RUSP disorders; providing training from other states with experience implementing new conditions; outsourcing second-tier testing to regional testing centers; providing technical assistance on new conditions through calls, webinars, and web-based resources; providing educational materials for healthcare professionals about new RUSP conditions; and creating a readiness tool for states to support the addition of new conditions to their panels. As one state NBS staff noted, “I don’t think we could do what we do [without these programs]. They’re the ones that help us move it forward. They connect us with other states. They provide us with screening algorithms…I would say these programs are our go-to on screening.”

Although progress has been made under this activity, gaps remain including insufficient federal guidance and funding for NBS implementation. This has resulted in a duplication of efforts across states and inequities in how differently states fund their NBS programs. Some participants wished for stronger guidance and consistent policies about adding RUSP conditions, as many were concerned by the variability in how quickly screening for new conditions are implemented across states. Participants also wished for more funding to help bolster NBS programs that may be at risk in some states and to help states add new conditions. Some worried about the security of federal public health funding. Additionally, participants mentioned the need for more collaboration among federal agencies and funded programs.

Participants also expressed concern over the NBS system’s capacity to handle new treatments and screening technology and the lack of infrastructure to enable data sharing, data standards, and harmonization across states. This includes data collected in the hospitals at the time of specimen collection, screening results stored in a Laboratory Information Management System (LIMS) database, short-term follow-up data located in electronic health records, and long-term follow-up data from registries and vital records. A few participants were familiar with recent federal investments in interoperability and data modernization and were hopeful about their potential impact.

### 3.2 Provide education, training, and TA on NBS to professionals

Participants noted that there is strong support for education and training opportunities for lab and follow-up staff, particularly around timeliness, adding new conditions, and a data repository on quality indicators. One state NBS staff commented that efforts to provide training, education and TA have been ongoing, saying, “It’s unprecedented the amount of time and energy spent, even during this past challenging year, to provide opportunities for people to enhance their skills and move forward with different projects, whether in the laboratory or with education and training.” In addition, funded programs have been particularly effective at providing education and TA to state NBS programs and labs and engaging NBS programs of all sizes. TA was provided in a variety of ways including a web site which hosted forums and a listserv, workgroups for states NBS staff on different topics, and individualized TA (both in-person and virtual).

However, additional training and TA is needed for state lab and follow-up staff around topics such as data analytics, adding new conditions, long-term follow-up, and communication between lab and follow-up staff. Participants noted that smaller states often need more TA than larger states, which tend to have more internal resources for training. These differences in resources dedicated to training and program improvement can deepen existing disparities between states. Participants suggested possible strategies to address this disparity, including funding to attend in-person training, provision of TA from contractors, and direct financial support for labs.

Participants noted that although trainings and education about NBS are available for healthcare professionals, there is not widespread awareness and engagement with these offerings. Participants noted the need for educating hospital staff who collect NBS specimens and communicating with families about the specimen collection process. Similarly, primary care providers, who are often tasked with delivering out-of-range NBS results to families, need education on understanding the NBS system, including what a positive screen means as well as how to communicate results to a family.

### 3.3 Establish follow-up and treatment for affected patients and their families

Short-term follow-up in the NBS system has been effective at connecting families of children with a positive screen to clinicians for confirmatory testing and diagnosis. Participants spoke to the helpfulness of a range of activities provided by federally funded program activities, including webinars and trainings on short-term follow-up, peer-to-peer learning across a variety of topics, and a data repository that collects data on quality indicators related to timeliness of short-term follow-up.

Despite successes with short-term follow-up, several participants emphasized the lack of focus on long-term follow-up. One of the key needs identified was a national long-term follow-up system that would enable states to track data on children with different conditions starting with confirmatory diagnosis and onward. This is especially important as new conditions are screened that may have less long-term evidence of treatment efficacy or variable phenotypes, such as late-onset conditions. As one subject matter expert said, “Sometimes you put a test on the panel and don’t have the data to know if you made a good choice or not and whether kids benefit… [What is needed is] a more robust national system to conduct research on these screening possibilities and develop registries and long-term follow-up so we can evaluate different treatment modalities and if kids are receiving benefit from the NBS.”

Additional concerns were expressed about the inconsistency in short-term follow-up with families after a positive screen. Follow-up processes vary among states and by condition, and often primary care providers who are contacting families are not familiar with the condition or that an out-of-range screen requires confirmatory testing. According to participants, providers need basic anticipatory training related to out-of-range screens and next steps. Participants also shared gaps related to connecting families with psychosocial supports after an out-of-range screen or diagnosis. More consistent systems are needed to connect families with appropriate resources, including educational materials, access to formula or other treatments, and social support. These psychosocial barriers are exacerbated when families do not have sufficient insurance to cover many of the services that are necessary to manage their child’s diagnosis. Collectively, these issues can result in inequitable access to confirmatory testing and treatment.

### 3.4 Improve the timeliness of NBS from specimen collection through diagnosis

Nearly all participants agreed that great strides have been made in improving the timeliness of NBS from specimen collection through diagnosis. It was noted that much of this progress was due to HRSA’s investments in establishing quality indicators and support to collect data and set a standard for states. One NBS program grantee said, “Sometimes just stating a goal makes people aim for that goal which before, they were just doing the best they could.”

Recommendations for future efforts included maintaining timeliness standards through ongoing education, quality improvement efforts, and funding. Participants indicated the timeliness needs to be consistently addressed due to staff turnover. Additionally, although the timeliness data includes quality indicators on reporting of time critical and non-time-critical screen-positive results, concerns were expressed about short-term follow-up quality indicators, including the amount of time from reporting screen-positive results to the receipt of medical intervention or a confirmed clinical diagnosis. This was likely due to the need to rely on healthcare providers, rather than NBS program staff, to collect the data. Several participants noted that timeliness may not need to be a focus for all conditions, and, in some cases, it may be more important to value precision in reporting data over speed.

### 3.5 Develop and disseminate educational materials

Several participants listed multiple programs that have increased the amount, quality, and availability of educational resources for families about staff. For example, several referenced a website which included easy-to-understand information on screening procedures, screening outcomes, and how to respond to screening results; a list of which conditions for which each state screens; disorder-specific information outlining early signs parents should look for, treatment options, and expected outcomes; and options for support services and how to access care. Participants appreciated the diversity of high-quality educational materials designed to reach a broad spectrum of families.

Participants stressed that reaching and educating families is a complex, multifaceted endeavor and requires a variety of educational strategies. As one healthcare provider noted, “Education isn’t one size fits all.” In general, there is the perception that there are a lot of good educational resources available to families, but that it is a very patchworked system with no centralized location for all materials and little collaboration among groups. The reach of educational materials often depends on partners, infrastructure, and funding, which is not consistent among states or conditions.

Other areas for improvement included that some educational materials are not updated as needed and often do not reach their intended audiences. Several participants noted that families turn to the internet and social media for disease-specific education and support, and there was some concern about the quality and accuracy of information available through general internet searches. To better reach families, participants suggested focusing on the timing of the dissemination of materials and providing more accessible and clear education in the prenatal period as a positive screen can be traumatic to families who lack awareness and may not understand the context. Additionally, participants noted the need for increased visibility for existing educational resources that may not currently be returned as the top results within Google or other search engines. Finally, parents stressed the need to target content appropriately. Many participants noted that families need education about a variety of topics in NBS, such as the difference between screening and a diagnosis.

### 3.6 Improve health equity and outcomes

Improvements in health equity and outcomes have been made through the support, guidance, and direction provided to the NBS system. The provision of education, training, and TA to enhance state capacity and support the implementation of new conditions has led to earlier diagnosis and treatment for all children. HRSA’s investment in a data repository that provides states with a system to track timeliness data and evaluate progress has significantly helped to improve the speed of receipt and screening of specimens and reporting results. In addition, the inclusion of medically underserved and diverse families in a needs assessment to determine the design and development of various educational materials has resulted in more inclusive resources for all families.

Participants shared diverse perspectives on the gaps in the NBS system related to health equity and outcomes. Many participants noted that the NBS system itself is a driver of equity since each baby receives the same screening, yet inequities emerge when states screen for different conditions, when timeliness becomes an issue, when families have challenges accessing confirmatory testing, and when families transition into clinical care and have unequal access to resources (e.g., specialized treatments, transportation, social and emotional support). These gaps in equity all are amplified by loss to follow-up, as access to care is diminished over time. Long-term follow-up is needed both to prevent loss to follow-up and to assess whether there are disparities in equity. These inequities are correlated with and driven by other well-known inequities in access to care such as systemic racism and implicit bias.

## 4 Discussion

### 4.1 Summary of findings

This evaluation provided important information on HRSA-supported federal investments in NBS. State programs have made several enhancements, with federal support to expand the number of conditions on state NBS panels and funding to provide technical assistance on the implementation of RUSP conditions among the most significant. Participants indicated that despite this success, there was a need to increase funding to states, especially those with fewer resources, to reduce the state-to-state variability in the types of conditions screened. In addition, participants called for more coordination among federal agencies. A strategic plan could be used to guide the future of NBS, with an emphasis on outlining how each agency aligned with the components of the plan. MCHB recently released a Blueprint for Change for all children and youth with special health care needs, which identified four key areas (equity, quality of life, access to services, and financing of services) and sets forth an agenda for the next 15–20 years (17). An NBS-specific plan would supplement this work. This would complement the National Academies of Sciences, Engineering, and Medicine consensus study report on NBS (18). This work may set the stage for a roadmap, similar to one that has been conducted for sickle cell disease (19).

Although state NBS programs have access to education and training resources on a variety of topics through federal programs, there are still gaps. In particular, participants mentioned several data-related needs, including support for data analysis and data management. With the likelihood of genomic sequencing coming to NBS, whether in the form of whole genome sequencing or targeted panels, these challenges will be exacerbated given the effort needed to process sequencing data, interpret variants, and store information long-term (20, 21). HRSA has addressed some of these needs with recent investments in interoperability (22). However, additional infrastructure support in informatics and clinical expertise to understand gene-disease pairs will be needed to move state-based NBS programs towards the genomic era.

Although many participants reported success resulting from efforts to improve the timeliness of NBS, most agreed that continued federal support is needed. Recent work provided data on quality improvement projects conducted by states to address various aspects of timeliness, from specimen collection, testing, or reporting out of results (23). States implemented different approaches depending on which timeliness indicator needed improvement, such as educational campaigns with hospital staff and birthing facilities, expanding operating hours or adding screening on weekends, or using health information technology to assist with data transfer. NBS programs will continue to need support to make improvements or maintain timeliness goals.

Participants also highlighted the need to educate healthcare providers (in particular, primary care providers), hospital staff, and families on the NBS system and specific conditions. Materials and trainings are often available and of high-quality, but dissemination has been challenging. Educational efforts have been hindered further by the workforce challenges in genetics (24). Earlier work with parents found a preference for communicating about NBS at multiple timepoints, beginning at preconception and continuing through the postnatal period (25, 26). A survey of prenatal providers indicated that although most agreed that NBS was important they thought either pediatricians or hospital staff would discuss it with women (27). Multimedia resources presented shortly after birth have been shown to increase parents’ understanding of NBS (28). Websites that provide information on NBS are variable in their quality (29). However, parents of children with an out-of-range screen often turn to the internet for information (30). Ensuring providers and families have timely access to high-quality NBS materials is essential for the continued success of this important public health program. Strong partnerships between NBS programs and a diverse group of system representatives can assist with successful educational efforts (31–33).

The lack of long-term follow-up data individuals diagnosed with conditions through NBS continued to be a noted gap. This echoes recommendations from other NBS experts who have long called for a need to focus on long-term follow-up data (34–36). HRSA has invested in long-term follow-up activities (37), however these were recently initiated programs that were not included in this evaluation. Work from these programs has resulted in further defining minimal data to collect on identified infants as part of clinical or public health long-term follow-up (38). Likewise, families have stressed the need for long-term follow-up data, especially related to communication and relationships with providers, roles of the care team and parents, and care access and utilization (39). These data would expand our understanding of who is receiving care or has been loss to follow up. This information is crucial to addressing health equity issues in access to clinical care and reducing disparities (40, 41).

### 4.2 Limitations

There are three limitations of our study that should be noted. First, although we included a diverse group, our participants consisted of a convenience sample. Further, we did not gather extensive demographic information on participants. Our findings, therefore, may not have been fully representative of all NBS participants. Second, we focused on specific initiatives and activities supported by HRSA. However, there are other federal agencies that support NBS programs that we did not cover in our evaluation. In particular, investments by the National Institutes for Health (e.g., rare disease research, pilot studies in NBS) and Centers for Disease Control and Prevention (e.g., support for implementation of new NBS conditions, NBS quality assurance program) were not included. Thus, we may not have captured all successes and gaps. Finally, although our use of the rapid turnaround analysis approach was appropriate for the evaluation and based on recommended practice, a traditional qualitative coding approach may have provided slightly different results.

## 5 Conclusion

NBS has maintained a strong tradition as a successful public health program (42). With emerging advancements and availability of genome sequencing and other technologies, the system is poised to undergo dramatic changes. Continued federal investments in state NBS programs are needed to provide training, TA, and education to enhance and expand screening capacity, conduct short- and long-term follow-up, and improve health equity and outcome for all children and their families.

## Data Availability

The raw data supporting the conclusions of this article will be made available by the authors, without undue reservation.

## References

[1] Watson MS Lloyd-Puryear MA Mann MY Rinaldo P Howell RR. Main report. Genet Med. (2006) 8:S12–S252. doi: 10.1097/01.gim.0000223467.60151.02

[2] Sontag MK Yusuf C Grosse SD Edelman S Miller JI McKasson S . Infants with congenital disorders identified through newborn screening—United States, 2015–2017. Morb Mortal Wkly Rep. (2020) 69:1265–8. doi: 10.15585/mmwr.mm6936a6, 32915168 PMC7499833

[3] Watson MS Lloyd-Puryear MA Howell RR. The progress and future of US newborn screening. Int J Neonatal Screen. (2022) 8:41. doi: 10.3390/ijns8030041, 35892471 PMC9326622

[4] Lloyd-Puryear MA Therrell BL Mann MY Eckman JR Telfair J. The role of the federal government in supporting state newborn screening programs In: Bailey MA Murray TA, editors. Ethics and newborn genetic screening: new technologies, new challenges. Baltimore, MD: Johns Hopkins University Press (2009). 178–94.

[5] McCandless SE Wright EJ. Mandatory newborn screening in the United States: history, current status, and existential challenges. Birth Defects Res. (2020) 112:350–66. doi: 10.1002/bdr2.1653, 32115905

[6] Stark Z Scott RH. Genomic newborn screening for rare diseases. Nat Rev Genet. (2023) 24:755–66. doi: 10.1038/s41576-023-00621-w, 37386126

[7] Bailey DB Porter KA Andrews SM Raspa M Gwaltney AY Peay HL. Expert evaluation of strategies to modernize newborn screening in the United States. JAMA Netw Open. (2021) 4:e2140998. doi: 10.1001/jamanetworkopen.2021.40998, 34964853 PMC8717100

[8] Newborn screening saves lives act of 2007, PL 110–204. Available online at: https://www.congress.gov/bill/110th-congress/senate-bill/1858/text (Accessed October 21, 2025).

[9] Newborn screening saves lives reauthorization act of 2014, PL 113–240. Available online at: https://www.congress.gov/bill/113th-congress/house-bill/1281/text (Accessed October 21, 2025).

[10] A guide to evaluation in health research. Available online at: https://cihr-irsc.gc.ca/e/documents/kt_lm_guide_evhr-en.pdf (Accessed October 21, 2025).

[11] Kidder DP. CDC program evaluation framework, 2024. MMWR Recomm Rep. (2024) 73:1–37. doi: 10.15585/mmwr.rr7306a1 PMC11444645 39316770

[12] Vindrola-Padros C Johnson GA. Rapid techniques in qualitative research: a critical review of the literature. Qual Health Res. (2020) 30:1596–604. doi: 10.1177/1049732320921835, 32667277

[13] Hamilton AB. Qualitative methods in rapid turn-around health services research. Seminar transcript, spotlight on women’s health. VA: Health Service Research and Development (2013).

[14] Richards KA Hemphill MA. A practical guide to collaborative qualitative data analysis. J Teach Phys Educ. (2018) 37:225–31. doi: 10.1123/jtpe.2017-0084

[15] Hamilton AB Finley EP. Qualitative methods in implementation research: an introduction. Psychiatry Res. (2019) 280:112516. doi: 10.1016/j.psychres.2019.112516, 31437661 PMC7023962

[16] Averill JB. Matrix analysis as a complementary analytic strategy in qualitative inquiry. Qual Health Res. (2002) 12:855–66. doi: 10.1177/104973230201200611, 12109729

[17] McLellan SE Mann MY Scott JA Brown TW. A blueprint for change: guiding principles for a system of services for children and youth with special health care needs and their families. Pediatrics. (2022) 149:e2021056150C. doi: 10.1542/peds.2021-056150C, 35642876

[18] National Academies of Sciences, Engineering, and Medicine. (2025). Newborn screening in the United States: a vision for sustaining and advancing excellence. Available online at: https://www.congress.gov/bill/117th-congress/house-bill/482/text (Accessed October 21, 2025). 40966374

[19] National Academies of Sciences, Engineering, and Medicine. Addressing sickle cell disease: a strategic plan and blueprint for action. Washington, DC: National Academies Press (2020). 33411430

[20] Remec ZI Trebusak Podkrajsek K Repic Lampret B Kovac J Groselj U Tesovnik T . Next-generation sequencing in newborn screening: a review of current state. Front Genet. (2021) 12:662254. doi: 10.3389/fgene.2021.662254, 34122514 PMC8188483

[21] Howard HC Knoppers BM Cornel MC Wright Clayton E Sénécal K Borry P. Whole-genome sequencing in newborn screening? A statement on the continued importance of targeted approaches in newborn screening programmes. Eur J Hum Genet. (2015) 23:1593–600. doi: 10.1038/ejhg.2014.289, 25626707 PMC4795188

[22] State newborn screening interoperability implementation program, notice of funding opportunity. Available online at: https://www.hrsa.gov/grants/find-funding/HRSA-21-085 (Accessed October 21, 2025).

[23] Sontag MK Miller JI McKasson S Sheller R Edelman S Yusuf C . Newborn screening timeliness quality improvement initiative: impact of national recommendations and data repository. PLoS One. (2020) 15:e0231050. doi: 10.1371/journal.pone.0231050, 32240266 PMC7117765

[24] Chung WK Dasgupta S Regier DS Solomon BD. The clinical geneticist workforce: community forums to address challenges and opportunities. Genet Med. (2024) 26:101121. doi: 10.1016/j.gim.2024.101121, 38469792

[25] Tluczek A Orland KM Nick SW Brown RL. Newborn screening: an appeal for improved parent education. J Perinat Neonatal Nurs. (2009) 23:326–34. doi: 10.1097/JPN.0b013e3181a1bc1f, 19915416 PMC2947955

[26] Araia MH Wilson BJ Chakraborty P Gall K Honeywell C Milburn J . Factors associated with knowledge of and satisfaction with newborn screening education: a survey of mothers. Genet Med. (2012) 14:963–70. doi: 10.1038/gim.2012.87, 22899093 PMC3908555

[27] Faulkner LA Feuchtbaum LB Graham S Bolstad JP Cunningham GC. The newborn screening educational gap: what prenatal care providers do compared with what is expected. Am J Obstet Gynecol. (2006) 194:131–7. doi: 10.1016/j.ajog.2005.05.075, 16389022

[28] Botkin JR Rothwell E Anderson RA Rose NC Dolan SM Kuppermann M . Prenatal education of parents about newborn screening and residual dried blood spots: a randomized clinical trial. JAMA Pediatr. (2016) 170:543–9. doi: 10.1001/jamapediatrics.2015.4850, 27043416 PMC7755042

[29] Araia MH Potter BK. Newborn screening education on the internet: a content analysis of North American newborn screening program websites. J Community Genet. (2011) 2:127–34. doi: 10.1007/s12687-011-0046-0, 22109819 PMC3186036

[30] DeLuca JM Kearney MH Norton SA Arnold GL. Internet use by parents of infants with positive newborn screens. J Inherit Metab Dis. (2012) 35:879–84. doi: 10.1007/s10545-011-9449-7, 22297410

[31] D’Silva AM Kariyawasam DS Best S Wiley V Farrar MA NSW SMA NBS Study Group . Integrating newborn screening for spinal muscular atrophy into health care systems: an Australian pilot programme. Dev Med Child Neurol. (2022) 64:625–32. doi: 10.1111/dmcn.15117, 34839535 PMC9299803

[32] Kemper AR Fant KE Clark SJ. Informing parents about newborn screening. Public Health Nurs. (2005) 22:332–8. doi: 10.1111/j.0737-1209.2005.220408.x, 16150014

[33] Davis TC Humiston SG Arnold CL Bocchini JA Jr Bass PF III Kennen EM . Recommendations for effective newborn screening communication: results of focus groups with parents, providers, and experts. Pediatrics. (2006) 117:S326–40. doi: 10.1542/peds.2005-2633M, 16735260

[34] Hinton CF Homer CJ Thompson AA Williams A Hassell KL Feuchtbaum L . A framework for assessing outcomes from newborn screening: on the road to measuring its promise. Mol Genet Metab. (2016) 118:221–9. doi: 10.1016/j.ymgme.2016.05.017, 27268406 PMC4970906

[35] Howell RR Engelson G. Structures for clinical follow-up: newborn screening. J Inherit Metab Dis. (2007) 30:600–5. doi: 10.1007/s10545-007-0674-z, 17694355

[36] Lloyd-Puryear MA Brower A. Long-term follow-up in newborn screening: a systems approach for improving health outcomes. Genet Med. (2010) 12:S256–60. doi: 10.1097/GIM.0b013e3181fe5d9c, 21150372

[37] Long-term follow-up for severe combined immunodeficiency and other newborn screening conditions, notice of funding opportunity. Available online at: https://www.hrsa.gov/grants/find-funding/HRSA-21-079 (Accessed October 21, 2025).

[38] Kellar-Guenther Y Barringer L Raboin K Nichols G Chou KY Nguyen K . Defining the minimal long-term follow-up data elements for newborn screening. Int J Neonatal Screen. (2024) 10:37. doi: 10.3390/ijns10020037, 38804359 PMC11130882

[39] Quesada S Barringer L Sontag MK Kellar-Guenther Y. A qualitative study on engaged families’ experiences with long-term follow-up care in the Colorado/Wyoming newborn screening system. Int J Neonatal Screen. (2024) 10:61. doi: 10.3390/ijns10030061, 39311363 PMC11417874

[40] Khoury MJ Bowen S Dotson WD Drzymalla E Green RF Goldstein R . Health equity in the implementation of genomics and precision medicine: a public health imperative. Genet Med. (2022) 24:1630–9. doi: 10.1016/j.gim.2022.04.009, 35482015 PMC9378460

[41] Ghaloul-Gonzalez L Parker LS Davis JM Vockley J. Genomic sequencing: the case for equity of care in the era of personalized medicine. Pediatr Res. (2025) 97:1393–8. doi: 10.1038/s41390-025-03869-6, 39843777

[42] Centers for Disease Control and Prevention (CDC). Ten great public health achievements—United States, 2001–2010. MMWR Morb Mortal Wkly Rep. (2011) 60:619–23. 21597455

