# Supplementary material for: An evaluation of U.S. federal investments in newborn screening: successes, gaps, and future directions
Source: Front Public Health. 2026 Feb 11;14:1729659. doi: 10.3389/fpubh.2026.1729659 (PMC12932493; doi:10.3389/fpubh.2026.1729659)
Supplement: Supplementary file 1 [file Supplementary_file_1.docx]

Interview Guide: HRSA NBS Evaluation

Stakeholder group: State and Local Health Departments

Interviewee name:

Welcome

Thank you for taking the time to speak with us today. My name is **[name]** and this is **[name].** We are with RTI International and are working with the Health Resources and Services Association (HRSA) to conduct an evaluation of HRSA’s portfolio of programs that focus on newborn screening. The purpose of the evaluation is to understand the needs of the newborn screening system from key stakeholders (such as you), the unique role HRSA’s programs play in addressing those needs, and unmet needs to inform future HRSA programs. The evaluation results will help set the stage for future newborn screening and genetics projects funded by HRSA, including the provision of technical assistance (TA), support, and education.

The purpose of our discussion today is to learn more about your experiences with the current newborn screening programs and your perspectives of the current and future needs of the newborn screening system. Our discussion should last approximately 60 minutes. If there are questions you don’t feel knowledgeable about or don’t feel comfortable answering, just let us know and we can move on.

Introduction

To get started, please tell us a little bit about yourself and your role within the newborn screening system.

Great, thanks for sharing.

Before we get started, I wanted to mention a few housekeeping items. We will be taking notes during our discussion but would also like to record the interview to make sure we capture everything correctly. The recording of this interview will not be shared outside the RTI research team, but the information gathered during this interview will be used in evaluation reports. However, we will not link your name to the information we collect. Do you give us permission to record the interview? Do you have any questions before we begin? Thank you.

**[BEGIN RECORDING]**

Background

**[Note: This information will be sent to participants ahead of time, so it might be okay to just say “Please refer to the document we sent you about the newborn screening system and HRSA’s goals, as needed, during today’s call”]** Before we dive into our questions, I want to provide some background information to set the stage for the discussion. When we refer to the newborn screening system today, we are referring to all steps in the system – from specimen collection through long-term follow-up – and all the people and systems involved in the newborn screening system, such as healthcare providers, families, lab and follow-up professionals, hospital staff, and information technology experts. Here is a graphic of the newborn screen system that we shared with you ahead of the call. [Show graphic]

*Adapted from www.aphl.org*

Our questions today will focus on the goals of the newborn screening system, how well the portfolio of HRSA-funded programs are meeting the goals, and the current and future needs of the newborn screening system. As outlined in federal legislation, the goals of the newborn screening system include:

- **Goal 1:** Enhance, improve, or expand the ability of state and local public health agencies to provide screening, counseling, and healthcare services to newborns and children having heritable disorders.
- **Goal 2:** Provide education, training, and TA to lab personnel and other genetics/health care professionals on the implementation of state-based public health newborn screening programs.
- **Goal 3:** Establish, maintain, and operate a system to assess and coordinate follow-up and treatment related to congenital, genetic, and metabolic conditions.
- **Goal 4:** Improve the timeliness of newborn screening from specimen collection through diagnosis.
- **Goal 5:** Develop and provide education to, and engage with, consumers (i.e., parents, families, patient advocacy groups) about screening, counseling, follow-up, and treatment to increase awareness, knowledge, and understanding of NBS and genetic conditions.
- **Goal 6:** Improve health equity and health outcomes of individuals with genetic conditions, reduce morbidity and mortality caused by genetic conditions (including congenital and metabolic disorders), and improve the quality of coordinated and comprehensive genetic services to children and their families.

Discussion Questions

OK, let’s get started. For our first set of questions, we will walk you through each of the goals of the newborn screening system and ask you how well you think the HRSA-funded programs are addressing these goals. We will start with: **Goal 2: Provide education, training, and TA to lab personnel and other genetics/health care professionals on the implementation of state-based public health newborn screening programs.**

1. On a scale of 1-5, how well do you think the portfolio of HRSA NBS programs is achieving this goal? (1 = *not at all,* 5= *very well)* Why did you choose this number? **(EQ1)**
2. What HRSA-funded programs, in particular, have been helpful in reaching this goal? *(Probe on NewSTEPs, Quality Improvement, Timeliness, Implementation of RUSP conditions)*
   1. **[Participants from states who were part of the State Evaluation programs]** How did HRSA’s NBS State Evaluation program funding help your state to achieve this goal?
3. Are there some areas of this goal that are being better addressed than others (e.g., lab staff versus health care professionals)? **(EQ2)**
4. What barriers are there in meeting this goal? **(EQ3)**
5. How would you evaluate the quality and impact of the program activities? **(EQ2)**
6. Are there additional HRSA activities or funded programs that address this goal? What other activities or programs should HRSA fund to help address this goal? **(EQ4)**

OK, let’s move on **to Goal 3: Establish, maintain, and operate a system to assess and coordinate follow-up and treatment related to congenital, genetic, and metabolic conditions.**

1. On a scale of 1-5, how well do you think the portfolio of HRSA NBS programs is achieving this goal? (1 = *not at all,* 5= *very well)* Why did you choose this number? **(EQ1)**
2. Which HRSA programs have addressed this goal?
3. What are some activities that these programs have implemented to reach this goal? Which of these activities have been the most helpful? **(EQ2)**
4. What are some barriers to addressing this goal? Do these barriers differ by state or NBS condition? **(EQ3)**
5. What other activities or programs should HRSA fund to help address this goal? *(probe on data systems and infrastructure, ability to track long-term outcomes)* **(EQ4)**

OK, let’s move on to **Goal 4: Improve the timeliness of newborn screening from specimen collection through diagnosis.**

1. On a scale of 1-5 (1= *not at all*, 5= *very well*), how well have HRSA’s portfolio of programs helped to improve the timeliness of NBS from specimen collection through diagnosis? Why did you choose this number? **(EQ1)**
2. One funded NBS program, in particular, has focused on Goal 4: Improving Timeliness of NBS Diagnosis program, also called NewSTEPs 360.
   1. How familiar are you with this program?
   2. What activities are you aware of that address timeliness in NBS? **(EQ2)**
   3. Are there some areas that are being better addressed than others (e.g., timeliness of spot collection, screening, report-out, diagnosis)? How would you evaluate the quality and impact of the program activities? **(EQ2)**
   4. Do you know of any challenges in meeting this goal? **(EQ3)**
   5. Are there additional activities or HRSA funded programs that address this goal? **(EQ4)**

Let’s move on to **Goal 5: Develop and provide education to, and engage with, consumers (i.e., parents, families, patient advocacy groups) about screening, counseling, follow-up, and treatment to increase awareness, knowledge, and understanding of NBS and genetic conditions.**

1. On a scale of 1-5, how well do you think the portfolio of HRSA NBS programs is achieving this goal? (1 = *not at all,* 5= *very well)* Why did you choose this number? **(EQ1)**
2. HRSA funds the NBS Family Education Program, which works to develop and deliver educational programs about NBS to parents, families, and patient advocacy groups. Are you aware of this program? What is your perception of how helpful this program is in meeting the state NBS family education needs? **(EQ2)**
3. What are your state’s needs and challenges in providing education on the NBS process or when working with families for follow-up? **(EQ3)**
4. What other activities or programs should HRSA fund to help address these needs? **(EQ4)**

OK, let’s move on to **Goal 1: Enhance, improve, or expand the ability of state and local public health agencies to provide screening, counseling, and healthcare services to newborns and children having heritable disorders.**

1. On a scale of 1-5, how well do you think the portfolio of HRSA NBS programs is achieving this goal? (1 = *not at all,* 5= *very well)* Why did you choose this number? **(EQ1)**
2. Over the past 5 to 10 years, what are the biggest improvements that have been made in the NBS system? How have HRSA programs worked to achieve these improvements?
3. How should HRSA measure success in reaching this goal? What metrics or indicators would you recommend? What other activities or programs should HRSA fund to help address this goal? **(EQ4)**

Finally, I’d like to talk about **Goal 6: Improve health equity and health outcomes of individuals with genetic conditions, reduce morbidity and mortality caused by genetic conditions (including congenital and metabolic disorders), and improve the quality of coordinated and comprehensive genetic services to children and their families.**

1. On a scale of 1-5 (1= not at all, 5= very well), how well do you think the portfolio of HRSA NBS programs is helping to improve health outcomes of individuals with genetic conditions, reduce morbidity and mortality, and improve the quality of coordinated and comprehensive genetic services? **(EQ1)**
2. What, if any, disparities exist in the NBS system and/or delivery of genetic services? How can these disparities be addressed to achieve health equity? *(Probe further about progress and goals as they relate to health equity)* **(EQ4?)**
3. What other activities or programs should HRSA fund to help address this goal? **(EQ4)**

Thanks for that helpful information. For the last set of questions, we would like to know more about the current and future needs of the NBS system.

1. What are the unmet needs or gaps that exist in the NBS system today?

- *Probe on training and technical assistance in genetic testing*
- *Informatics support, collaboration with clinicians on follow-up)*? How can HRSA provide funding and support to meet these needs or address these gaps? **(EQ5)**

1. What are your specific state needs and what can HRSA do to help address those needs? **(EQ4)**
2. What, in particular, are your needs as a state lab or follow-up personnel as you look to the future of NBS? How can HRSA provide funding to be better prepared to meet these needs? **(EQ6)**

Closing

Thank you so much for taking the time to tell us about your experiences with the NBS programs. Before we end our call, is there anything else you’d like to share or anything you thought I should have asked during this interview but may have missed?

You will receive an Amazon gift card for your participation today. Thank you!

**[END INTERVIEW]**
